# Four Haylages, One Metabonome: Defining Diet Driven Metabolic Shifts in Ponies

**DOI:** 10.1111/jpn.70099

**Published:** 2026-08-06

**Authors:** Meriel Moore‐Colyer, Pat Harris, Simon Daniels

**Affiliations:** ^1^ Deparment of Animal Health Hartpury University Gloucester Hartpury UK; ^2^ Equine Studies group, Waltham Petcare Science Institute Mars Horsecare, Waltham–on–the–Wolds Leicestershire UK; ^3^ Royal Agricultural University, Stroud Road Cirencester UK

**Keywords:** faecal, haylage, horse digestion, metabolomics, urine

## Abstract

Haylage is widely fed to all categories of stabled horses across Europe and New Zealand. Haylage can vary from high energy nutrient dense forage to low energy highly fibrous fodder, yet little is known about the impact different haylages have on metabolism in horses. This study provides the first direct comparison of feeding four different haylages: early cut (*Lolium multiflorum*), later cut (*Lolium perenne*) rye grass (R1 and R2); early and late cut meadow grass (M1 and M2), on urinary and faecal metabonomics, in ponies at maintenance. Using replicated Latin Square design, eight individually housed ponies were fed each haylage for 15 days in four periods. Faeces and urine were collected on day 12 for metabonomic profiling. Apparent digestibility was evaluated on 3 days of total faecal collection (days 10–12) before adaptation to the new diet (days 13–15). Metabolic profiles (^1^H NMR Spectroscopy), were phased and baseline corrected via TSP singlet δ 0.0. Principal Component Analysis (PCA) via Matlab, was used to identify metabolic profiles and Orthogonal Projections to Latent‐Structures Discriminate Analysis (OPLS‐DA) models to group specific metabolic changes. Digestibility was determined using multivariate mixed effects modelling +ANOVA. Pre‐study urinary metabolic profiles were similar for all ponies. R1 haylage produced differentiating urinary clusters relative to R2 and M1 although no individual metabolites differed significantly. Some faecal R1 clustering was evident. Pairwise metabolites comparisons reflected underlying nutrient profiles. High inter‐pony variation did not prevent dietary discrimination, with R1 > M1 > M2 = R2. This study provides the first evidence that haylages differing in maturity and botanical composition generate measurable metabolic shifts in ponies. These findings highlight the biological importance of haylage selection and indicate that metabonomic profiling is a sensitive informative tool for assessing how forage characteristics shape digestive physiology.

## 1 Introduction

It is now well accepted that feeding high‐forage diets are preferable to high‐starch diets for maintaining digestive, metabolic, developmental and behavioral health in horses (Müller 2012; Galinelli et al. 2021; Hockenhull and Creighton 2014). The choice of which forage to feed is often determined by price and availability (Moore‐Colyer et al. 2023) although nutrient content and hygienic quality are also important factors (Ragnarsson and Lindberg 2010; Müller 2012). Increasing knowledge about the high respirable dust content of hay has persuaded many owners of horses with respiratory disorders, to make haylage their preferred forage (Leng et al. 2021). Haylage is widely fed across Northern Europe but can range from young leafy grass that has been conserved by lactic acid fermentation and generally has a high digestible nutrient content, to a more mature high fibre forage with a lower digestible nutrient content which has been conserved by anaerobic conditions (Harris et al. 2017).

Jansson and Lindberg (2012) demonstrated that feeding a nutrient‐dense (10.4 ME/kg dry matter (DM) and 104 g CP/kg DM) early cut Timothy and Meadow Fescue haylage with a forage balancer provided sufficient nutrients to maintain high work rates in exercising trotting horses, thus demonstrating the benefits of sourcing high‐quality haylage for working horses. However, energy dense haylage is not suitable for all horses, particularly non‐working animals or those with metabolic disorders related to obesity that are on restricted dietary intake regimens (Argo et al. 2012). For these animals a more fibrous, lower nutrient‐content haylage may help to maintain eating times without supplying excess calories, although restricting daily intake may still be necessary as obesity can result from forage‐only diets (Dugdale et al. 2011).

Whatever type of haylage is fed, it is important that it maintains a healthy hind gut microbiome. Feeding high‐forage diets to adult horses has long been associated with eubiosis, supporting a more resilient mucosal barrier and reducing susceptibility to dysbiosis (Dougal et al. 2014; Julliand and Grimm 2017). In young, growing horses, high‐fibre rations have been shown to improve temperament and reduce lactic‐acid production, a marker of dysbiosis, when compared with high‐cereal diets (Moore‐Colyer et al. 2020; Park et al. 2021). The influence of the microbiome on temperament has also been demonstrated in adult horses (Bulmer et al. 2019; Destrez et al. 2019). Destrez et al. (2019) reported that horses fed all‐fibre diets, as opposed to high‐starch diets, exhibited microbiota shifts that were closely linked to reduced anxiety‐related behaviours during stress tests. Across two behavioural paradigms, the horses' responses correlated strongly with microbial signatures characteristic of high‐fibre versus high‐starch feeding. Such studies highlight the role of the gut‐brain axis in horses and demonstrate that diet‐driven alterations in the gut ecosystem can directly influence behavioural reactivity under stress.

Forage maturity has been noted to have a major impact on gut microbial profiles (Ragnarsson and Lindberg 2010; Ragnarsson and Lindberg 2008), with Müller (2012) attributing this to the forage lignin content which, together with cellulose seemed to negatively impact fermentation by interfering with the microbiome‐encoded carbohydrate active enzymes (Li et al. 2015). Therefore, forage quality, alongside animal factors, management regimens, stress levels and training regimens contribute to the differing gut microbiome profiles noted in horses in previous studies (Bulmer et al. 2019; Dougal et al. 2017; Gomez et al. 2021; Morrison et al. 2020). Weinert‐Nelson et al. (2023) reported that non‐structural carbohydrate and crude protein contents in warm and cool season grasses influenced the faecal microbiota and metabolic responses in horses, but to date how different haylage types and maturity impact the equid gut is poorly understood.

The equid gut, in common with other mammals, has thousands of different microbes within the gut ecosystem, with many performing similar actions (Dougal et al. 2013). Therefore, some inter‐animal variability is inevitable and may be inconsequential to overall gut function and health as functional redundancy is likely to occur (Morrison et al. 2020; Daniels et al. 2025). Thus, examining the output of fermentation products available for metabolism, rather than the microbiome itself, may be more informative when determining the impact of diet changes on the horse.

Metabonomic studies monitoring the effect of medication (Arnold et al. 2020), exercise (Bazzano et al. 2020; Johansson et al. 2024), and diet (Zhu et al. 2021) on salivary and serum metabolites in horses have been reported, but to the authors' knowledge no studies have been reported on the effect of different haylages on urinary and faecal metabonomics in ponies. Leng et al. (2021) used urinary metabonomics to monitor the effect of feeding hay vs haylage, reporting that hay‐fed animals had higher Hippurate (indicative of a greater microbial diversity and a healthy gut) compared with feeding haylage, which produced higher levels of ethyl‐glucoside (a compound found in wine and sake and indicative of fermentation). Although no information was given on the nutrient profiles of each forage, they were conserved at different stages of growth, suggesting that forage physiological age may have had an impact on metabonomic output.

Therefore, the aim of this study was to investigate the effects of feeding four haylages differing in physiological maturity and grass composition on the urinary and faecal metabolic profiles in eight ponies at maintenance. It was hypothesized that the younger, more vegetative forages would be more digestible and produce more propionate and metabolites associated with protein digestion, while the more mature fibrous forages would be less digestible and produce more acetate. It was also hypothesized that despite feeding a high‐energy forage, no metabolites associated with poor gut health would be detected. As forage is such a key component of the equid diet, and plays a major role in maintaining gut health, influencing physiological factors via the gut‐brain axis providing resilience against stress, this first demonstration of how different haylages impact equid gut physiology is critically important in order to expand our knowledge of how feeding practices are reflected in metabolic activity and may impact gut health in horses.

## 2 Materials and Methods

### 2.1 Study Design

Eight adult Welsh Mountain ponies, with no recent history of structured exercise, with an average body weight (BW) of 264 kg (ranging from 217 to 305 kg), mean age 9 years (± 2.1) were fed four different haylages over four periods in a replicated Latin Square design trial, with pony, feed and period as factors. The four periods, which were of 15 days duration, were divided into two sections; 12 days where ponies were fed 100% of their allocated haylages and then 3 days i.e., day 13, 14 and 15 when they were changed over to the new haylage gradually i.e., old: new 70:30, old: new 50:50 and old: new at 30:70. Collections for metabonomic measurements were made on day 12 when ponies had spent the maximum time on a single diet. Collections were taken at 06.30, 10½ hours after the evening hay net had been given and before the morning feed at 08.00. Apparent digestibility was determined from collections made in the last 3 days (10, 11, 12) of each period. The period duration was based on common management systems where large baled (300–500 kg) HY is fed for 10–14 days before a new bale is opened thereby enabling the main aim of the study to be addressed i.e., to measure the effect of changing between different haylages on gut metabonomics in ponies). Although 10 days is shorter than is often employed for apparent digestibility assessment, previous work (Goachet et al. 2009; Hughes et al. 1995; Bockisch et al. 2023) showed that shorter adaptation durations yielded accurate digestibility measurements which was deemed acceptable for this part of the study.

The trial was carried out in accordance with the EU standards for the protection of animals, at the Royal Agricultural University Equestrian Centre. The study protocol was approved by the Royal Agricultural University Ethics Review Committee number 20204603‐Daniels. The trial was 3 months long and started on 19th October 2021 and finished on 18th December 2021.

### 2.2 Study Diets

The HY were all made by a commercial producer (Country Haylage, Cherry Orchard Farm, Youngwood Ln, Nailsea, Bristol BS48 4NP UK). The meadow haylage fed in the pre‐trial 10 days (P0) was conserved from a different area of the farm to that used to produce the trial haylages. This basal meadow haylage was harvested on the 8th of July 2021 and had a chemical composition of: DM 848 g/kg, pH 5.91 CP 51.1 g/kg DM, NDF 547 g/kg DM, ADF 329 g/kg DM. The four trial HYs were from 2 different pasture types and were conserved on different dates. The rye grass pastures were *Lolium multiflorum* (R1) and *Lolium perenne* (R2) and the meadow pastures a mix of *Lolium perenne*, *Festuca rubra, Poa trivialis* and *Holcus lanatus*. The two adjacent meadow grass fields used to produce M1 and M2 were managed similarly in every way except for cutting date. M1 was in a largely vegetative state, although some grasses showed early anthesis, and was conserved on 2/6/21; M2 was in full anthesis and was conserved on 8/7/21. The R1 was conserved on 24/5/21 and cut in a vegetative state, while R2 was the most mature haylage and was cut 28/7/21 when at full anthesis.

All haylages were originally made into large 600 kg square bales wrapped in black plastic before being stored in large stacks in the yard. The early cut haylage, R1 and M1 were stored in these big bales for approx. four months and the later cut M2 and R2 for 3 months before being processed into small bales of approx. 20 kg each on 5/10/21. The small 20 kg bales were made by opening each large square bale and feeding it through a large commercial small baler on Country Haylage premises. As part of the process, the small bales were vacuum packed into thick‐gauge white plastic bags and clearly labelled with type, big bale number and date. Bales were then transported by road to the study site and stored on wooden pallets in a covered barn until they were opened and fed to the ponies.

### 2.3 Animal Management

Ponies were managed as per the protocol detailed in Daniels et al. (2024). All ponies were deemed to be in good health from veterinary assessment at the start of the study and had been maintained on a regular healthcare programme including endoparasite control and dental maintenance. To acclimatize the ponies to a conserved diet and the slightly more restrictive regimen of being housed, all 8 ponies were brought in from pasture and managed according to the below protocol for 10 days (period 0) prior to the commencement of period 1. During this time all ponies were fed a basal diet of meadow grass haylage. Ponies were weighed on an equine appropriately calibrated weighbridge at the beginning of each period and then fed at 1.75% BW in DM/day; As most owners feed their horses by weight (Moore‐Colyer et al. 2023) it was decided to replicate industry practice as far as possible so diets were fed on a % body weight in DM per day. This meant that, as occurs in industry when owners change between forages, ponies received significantly more DE/day when consuming the R1 haylage compared with the other three haylages.

Ponies were housed in 12.2 m^2^ loose‐boxes with rubber mat/comfort stall (Haygain Ltd) flooring with ¼ of the area covered with shavings to absorb urine. Water was provided ad libitum from 15 litre tubs. The haylage was given in four equal meals at 08.00, 12.00, 16.00, and 20.00, in small‐holed hay nets. During non‐collection days exercise was provided twice daily in the form of ‘free group play’ for 30 min in a dry‐lot area of ~12 × 12 m, or on a horse walker. During data collection days, observed exercise occurred in pairs in the dry lot area and any faecal outputs were collected and recorded. Faecal samples for apparent digestibility measurements were taken on days 10, 11, 12 via total collection. Faeces were collected for each 24 h period into large bins before being thoroughly mixed and a sub‐sample taken for analyses.

### 2.4 Feed and Faecal Analyses

Haylage feed samples were collected from freshly opened individual 20 kg bales from five different areas and pooled for laboratory analysis. pH was read using a Thermo Scientific Orion Star A211 meter after 25 g of chopped haylage had been soaked at room temperature in 100 ml of de‐ionised water. Feed and faecal samples were dried at 60°C in a force‐draught oven until they reached constant weight to establish dry matter (DM) and then milled in a Fritsch P19 cutting mill and standard cyclone system with a two mm sieve. Milled samples were then analyzed according to the Association of Official Analytical Chemists International (2012), for organic matter (OM) Method 942.04, acid detergent fibre (ADF), Method 973.18 and neutral detergent fibre (NDF) Method 2002.04. Crude protein (CP) was determined using the Kjeldahl process; gross energy (GE) via bomb calorimetry in a Parr 6100 calorimeter.

Water‐soluble carbohydrate content (WSC) of the haylages was determined using the phenol‐sulfuric acid method as follows: 200 mg of dried, milled sample was placed into 35 ml of distilled water in 250 ml pre‐warmed (40°C) conical flask in a shaker incubator. Samples were shaken at 100 rpm for 1 h. one ml of sample was pipetted into microtubes and centrifuged at 12,000 × g for 10 min at room temperature. Samples were then diluted by 25% by transferring 250 ul to a clean microtube and 750 ml distilled water added. Tubes were mixed by inversion. 100 ul of diluted sample was then placed into designated wells on a 96‐well plate where 30 µl of phenol and 150 µl of sulphuric acid were added and the plate incubated at 90°C for 5 min in a water bath. Following incubation concentrations of WSC were read at 490 nm against prepared standard solutions of 1:1 Fructose: Glucose monohydrate (Dubois et al. 1956; Masuko et al. 2004).

### 2.5 Faecal and Urinary Sample Collection for Metabonomics

Sampling was undertaken in the mornings at the end of the adaptation P0 diet, and then on the morning of day 12 of each feeding period. Faecal water samples for pH analyses and metabonomics were taken from fresh spontaneously voided hand‐squeezed faeces. All faecal samples were collected in triplicate into 2.0 ml sterile tubes and placed immediately on ice for transportation. Urine samples for metabonomics were collected immediately from spontaneously voided urine. Urine was collected into deionized water washed sterile collection beakers attached to a handle and transferred in triplicate into 5 ml sterile cryovials and placed immediately on ice for transportation to the laboratory. All collected samples for metabolomics were frozen at −80°C within 3 h of collection.

### 2.6 Metabolic Profiling of Samples

Untargeted metabolic profiles were measured by ^1^H NMR spectroscopy in urine and faecal water extracted via centrifugation from the collected faecal samples. Samples were defrosted, then diluted with a phosphate buffer with Sodium 3 Trimethylsilyl Propionate‐d4 (TSP) as an internal standard, as described by Escalona et al. (2015). Briefly the urine samples were centrifuged at 10,000 × *g* before dilution with the buffer, 400:200 µl respectively. To create faecal water 0.3 g faeces and 1.2 ml of buffer were bead broken for 10 min and then centrifuged at 16,000 × g for 10 min three times, after each centrifugation the supernatant was removed and placed in a new microfuge tube before centrifuging again to remove the debris. From the diluted samples, 550 µL was pipetted into 5 mm NMR tubes.

The samples were analysed on a Bruker Avance NEO 600 MHz equipped with a TCI Cryoprobe Prodigy (Bruker). Spectra were acquired at 298 K and consisted for each sample of a ^1^H Carr‐Purcell‐Meiboom‐Gill (CPMG) spectrum, with 64 scans, an acquisition time of 2.62 s, a relaxation time of 4 s, a total spin echo delay of 30 ms and a spectral width of 20.8 ppm. After acquisition, the spectra were then phase corrected, baseline corrected, and the chemical shifts were referenced to the Trimethyl Silyl Propionate (TSP) peak at 0.0 ppm. For metabolite identification, 2D spectra were acquired, consisting of a Total Correlation SpectroscopY (TOCSY), using the Bruker pulse sequence “dipsi2gpphzs”, slightly modified to include presaturation, which 8 scans, 256 t_1_ increments, a spectral width of 13.7 ppm in both dimensions and a relaxation delay of 2 s, along with a Heteronuclear Single‐Quantum Correlation (HSQC) spectrum, using the Bruker pulse sequence “hsqcetgpsisp2.2”, with 16 scans, 256 t_1_ increments, a spectral width of 210 ppm in the ^13^C dimension and 20.8 ppm in the ^1^H dimension and a relaxation time of 1.75 s. Alignment and normalisation (total quotient method) of spectra was carried out in Mathworks Matlab R2014a.

### 2.7 Data Analyses

Metabonomic data were analysed initially by unsupervised principal component analysis (PCA) models. Where spatial clustering between groups occurred, supervised models were constructed using orthogonal projections to latent‐structures‐discriminate analysis (OPLS‐DA). OPLS‐DA models were used to identify group‐specific metabolic changes. Within OPLS‐DA models, goodness of fit is measured by R^2^Y and prediction ability is measured by Q^2^Y. Only OPLS‐DA models with Q^2^Y values > 0.4 and individual metabolite abundances differences R^2^ > 0.4 are reported. It is possible with these complex models that over‐fitting occurs, where too many latent variables are added reducing the predictive ability and creating bias within the model Gowan et al. (2010). To ensure models were not over‐fitting, OPLS‐DA models underwent permutation testing, to 1000 permutations to ascertain the significance by comparing the permutated model to the original OPLS‐DA model. Only models with good predictive ability and no over‐fitting (*p* < 0.01) were kept. Where models were kept, and differing metabolites were observed, concentrations were predicted by measuring the peak area and number of protons against that and the concentration of the TSP singlet.

Apparent digestibility data were subjected to normal distribution analyses via Shapiro‐Wilk test, followed by a multivariate mixed‐effects model analyses (REML, Genstat 22nd 2019) to take account of biological relationships between parameters and capture the nested structure of the data, reduce bias and improve power. FDR (Benjamini‐Hochberg) post hoc pairwise testing and finally individual ANOVAs (Genstat, 22nd 2019) were performed with pony (8), haylages (4) and period (4) as fixed factors. Differences between means were determined using Tukeys HSD post *hoc* test and significance was set at *p* < 0.05. Relationships were explored between variables (presented in supplementary data S4) Pearson's correlations were undertaken. Apparent digestibility (g/kg) was determined using the following standard equation:

ChemicalContentinfeed(g)×feedintake(g)–chemicalContentinfaeces(g)×faecaloutput(g)/chemicalContentinfeed(g)×feedintake(g)

Digestible energy content of feeds (DE MJ/kg DM) was calculated using the equation below from gross energy (GE) as determined using a bomb calorimeter, and GE digestibility:

DE=gross energy digestibility (GED)×GE content of feed

DE=digestible energy intake per day (DEI MJ/day)=Feed DE content (MJ/kg DM)×DMI

## 3 Results

All ponies remained healthy for the duration of the trial. All diets were readily consumed and negligeable feed refusals were collected across all periods. BW remained stable (±6.98 kg) throughout the trial. In this study day 12 was considered to represent fully stabilised hindgut fermentation for all ponies on all four haylages, as urinary and faecal metabonomic profiles did not differ between samples collected after 6 days of adaptation to a new diet (reported in a separate manuscript) and those collected on day 12.

### 3.1 Haylage Nutrient Content

The nutrient profiles of each of the four distinct haylages are shown in Table 1. Physiological age and haylage type influenced nutrient content with the R1 haylage being notably different from the other 3.

**Table 1:** Mean (+/−SD) chemical, energy and pH contents of four haylages Meadow haylage 1 (M1), Meadow haylage 2 (M2), Rye grass haylage 1 (R1) and Rye grass haylage 2 (R2) fed to 8 ponies in a replicated Latin Square trial.

Parameter	M1	M2	R1	R2
Conservation date	2/6/21	8/7/21	24/5/21	28/7/21
pH	5.80 (±0.09)	5.91 (±0.19)	5.67 (±0.10)	5.96 (±0.09)
Dry Matter g/kg	768 (±42.9)	800 (±54.8)	597 (±38.8)	822 (±15.3)
Neutral Detergent Fibre (g/kg DM)	583 (±37.0)	589 (±25.0)	402 (±122.)	562 (±16.5)
Acid Detergent Fibre (g/kg DM)	306 (±27.00)	347 (±28.60)	193 (±7.60)	347 (±19.64)
Crude Protein (g/kg DM)	75 (±4.49)	47 (±5.90)	67 (±5.75)	63 (±5.36)
Water‐Soluble Carbohydrates (g/kg DM)	156 (±53.54)	169 (±14.89)	411 (±16.30)	214 (±14.39)
Gross Energy MJ	17.6 (±0.09)	17.8 (±0.20)	16.5 (±0.33)	17.2 (±0.36)
Digestible Energy MJ/kg DM	13.5 (±1.64)	11.5 (±1.28)	15.3 (±1.84)	12.3 (±1.41)

### 3.2 Urinary Metabonomic Profiles

The urinary metabonome was explored using unsupervised PCA analyses after feeding each haylage for 12 days and the pre‐trial basal meadow haylage diet, which was fed for 10 days in P0 (Figure 1). The basal meadow haylage fed to all the ponies at the same time showed a clear and differentiating cluster, which separated this haylage from the four trial diets. Apparent digestibility measurements were not determined for this basal haylage. Multivariate analyses produced a tighter cluster for the R1 haylage compared with the other 3 trial haylages. However, the R1 cluster was surrounded by the profiles from the other 3 haylages, and thus all 4 trial diets produced similar metabonomic phenotypes. This is mostly reflected in the apparent digestibility data, which shows a high degree of similarity across all four haylages for all but DMD, where the R1 haylage was more digestible than the other three diets (Table 2).

**Figure 1:**
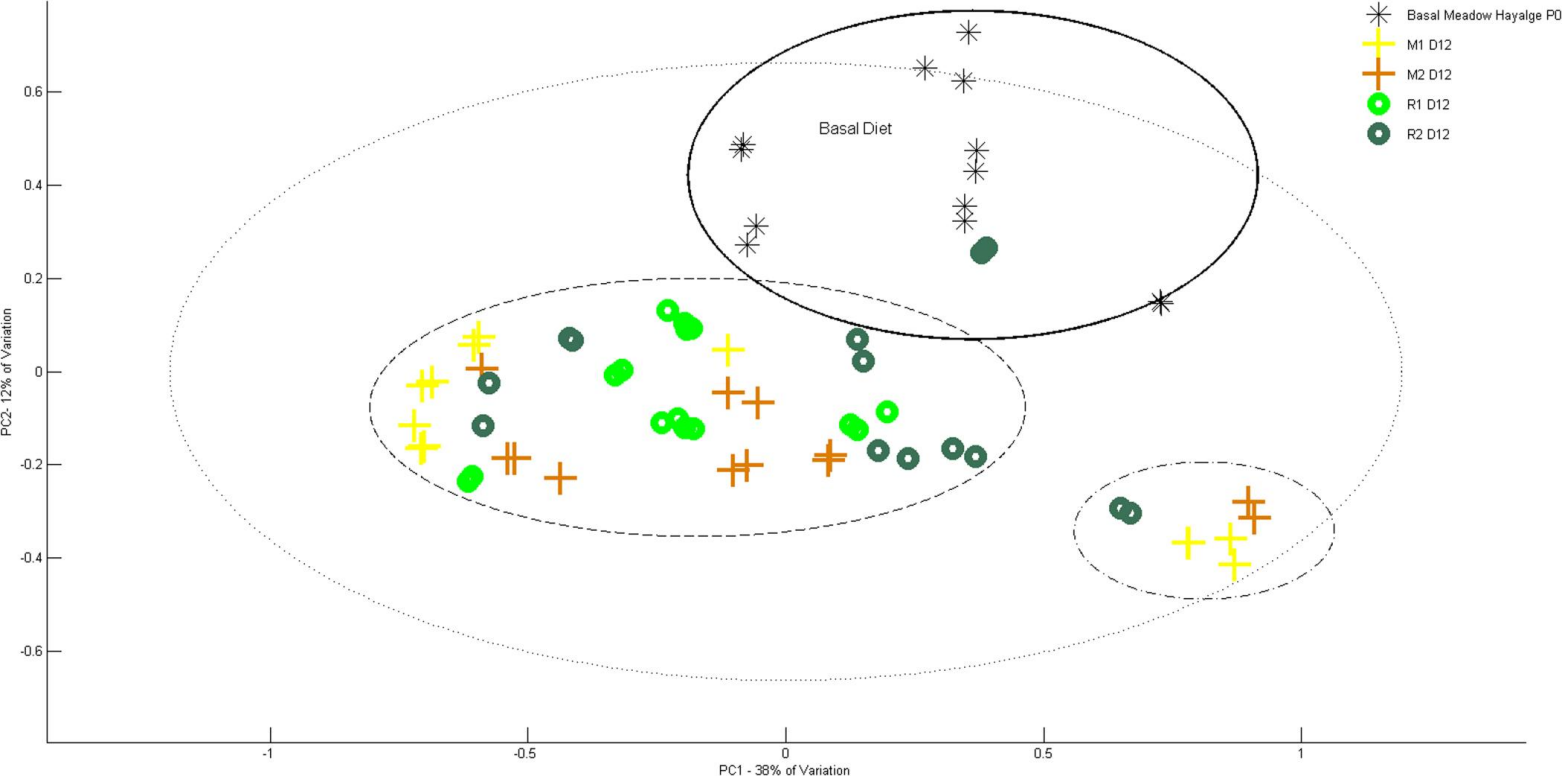
Principal component analyses (PCA) of the urinary metabolic phenotypes from eight ponies fed five different grass haylages. [Color figure can be viewed at wileyonlinelibrary.com]

**Table 2:** Mean apparent digestibility (± SE) and faecal pH of 8 ponies when fed Meadow haylage 1 (M1), Meadow haylage 2 (M2), Rye grass haylage 1 (R1) and Rye grass haylage 2 (R2).

	M1	M2	R1	R2	sed	Sig
Faecal pH	6.06	6.13	6.02	6.42	0.146	0.06
Dry Matter Digestibility g/kg	647^b^ (±9.4)	577^a^ (±12.9)	713^c^ (±30.5)	587^a^ (±22.9)	21.4	0.02
Crude Protein Digestibility g/kg	580^b^ (±19.0)	438^a^ (±21.0)	464^a^ (±57.5)	543^ab^ (±27.2)	41.1	0.00
Neutral Detergent Fibre Digestibility g/kg	781 (± 20.2)	781 (± 20.9)	752 (± 37.0)	785 (± 22.8)	34.7	NS
Acid Detergent Fibre Digestibility g/kg	605^ab^ (±24.0)	552^a^ (±47.6)	696^b^ (±27.2)	503^a^ (±47.2)	44.6	0.00
Digestible Energy intake MJ/d	54.9^a^	51.5^a^	68.5^b^	54.2^a^	3.9	0.00

^abc^values in the same row sharing the same letters are similar (*p* > 0.05).

A small cluster was observed in the positive lower quadrant for four ponies when consuming M1, M2 and R2. This smaller cluster represented four ponies, and occurred only in periods one and two; in periods three and four the metabolic phenotypes of all the ponies were similar.

When comparing pony responses to each diet using pair‐wise analyses, some clustering was seen as shown in Figure 2. Eg between R1 vs. R2 and R1 vs. M1. Clustering between the other three HY was less evident and did not show any differentiation between diets for M1, M2 and R2.

**Figure 2:**
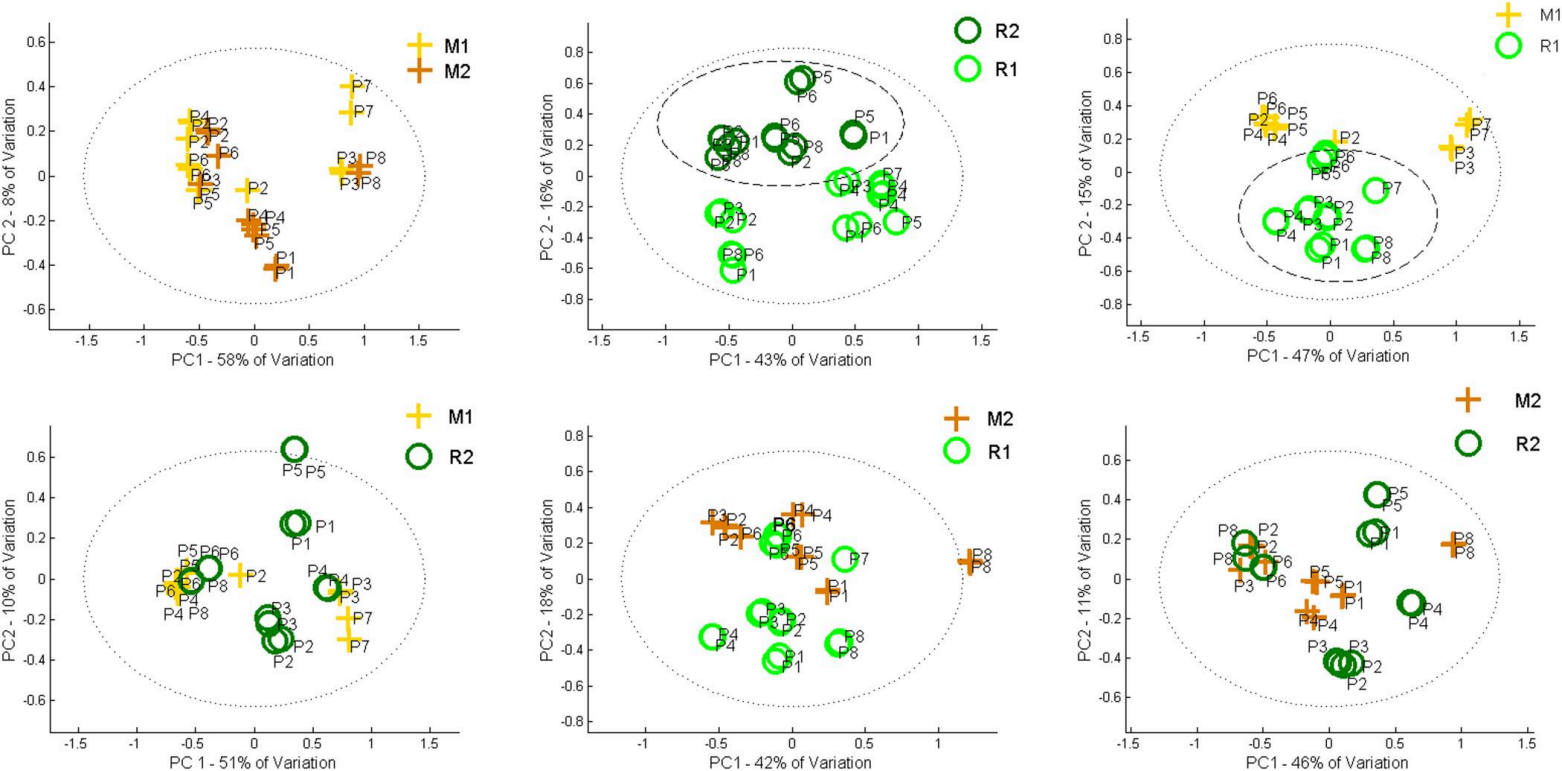
Panel plot of principal component analysis of urinary metabolic phenotypes for each diet in a pairwise comparison with each pony labeled to show individual animal effect. [Color figure can be viewed at wileyonlinelibrary.com]

Where metabolite differences were detected in the OPLS‐DA models, estimated concentrations were calculated against the TSP standard. These individual metabolites are detailed in Table 3 and show when comparing the urinary metabonomes between the four haylages, that there were three models (M1 vs. M2, M1 vs. R1 and R1 vs. R2) with acceptable predictive ability. However, the fact that no differences were noted between haylages for any individual metabolite suggests that separation between diets was mainly driven by multivariate analyses rather than differences in individual urinary metabolites. For each of the other pairwise comparisons, the models produced poor predictive ability (Q^2^Y < 0.4) or were overfitting latent variables to the model. However, when these estimated metabolite concentrations were compared between the haylages diets there were no differences in concentrations for any of these comparisons (*p* > 0.05).

**Table 3:** Urinary metabolites from 8 ponies when fed diets of Meadow 1 (M1) Meadow 2 (M2), Ryegrass 1 (R1) and Ryegrass 2 (R2).

	Q^2^Y & Permutation *P* values	Metabolite	^1^H Resonance (δ)	Correlation coefficient (R^2^)	Predicted concentration (Mean + SD) (μM)	Concentration probability value	Function & reference to the human/livestock metabolome database
**M1 V M2**	Q^2^Y 0.487 *p* = 0.033	Propionyl carnitine	2.46 (q)	0.6	M1 74.61 ± 153.93 M2 86.72 ± 133.19	*p* = 0.51	LMDB00253 Acyl carnitines
**M1 V R1**	Q2Y 0.695 *p* < 0.001	Propionyl carnitine	3.19 (s)	0.75	M1 52.31 ± 128.94 R1 26.16 ± 60.99	*p* = 0.46	LMDB00253 Acyl carnitines
		Propionyl carnitine	2.46 (q)	0.7	M1 23.08 ± 21.29 R1 15.13 ± 133.08	*p* = 0.83	LMDB00253 Acyl carnitines
**R1 V R2**	Q^2^Y 0.826 *p* < 0.001	Dihydroxyacetone	4.42 (s)	0.65	R1 38.92 ± 137.99 R2 70.02 ± 123.57	*p* = 0.38	HMDB0001882 Glycerol metabolism
		Dihydroxyacetone	4.43 (s)	0.6	R1 35.23 ± 129.29 R2 58.80 ± 143.77	*p* = 0.51	HMDB0001882 Glycerol metabolism
		Dimethylamine	2.72 (s)	0.6	R1 23.65 ± 83.28 R2 48.11 ± 70.37	*p* = 0.37	LMDB00037 Dimethylamine is abundantly present in animal urine and a precursor or TMAO
		Isoleucine	1.26 (m)	0.6	R1 27.83 ± 110.96 R2 35.46 ± 84.97	*p* = 0.59	LMDB00077 Branched chain amino acid
		Isoleucine	1.97 (m)	0.65	R1 23.95 ± 63.23 R2 38.84 ± 80.77	*p* = 0.91	LMDB00077 Branched chain amino acid
		O‐Succinyl homoserine	2.06 (m)	0.6	R1 97.92 ± 375.32 R2 43.91 ± 54.46	*p* = 0.37	HMDB0255868 Belongs to the class of organic compounds known as alpha amino acids
		TMAO	3.27 (s)	0.7	R1 12.89 ± 56.05 R2 28.46 ± 48.84	*p* = 0.31	LMDB00278 Trimethylamine N‐oxide (TMAO) is an oxidation product of trimethylamine and a common metabolite in animals. Gut bacteria (e.g., species of Acinetobacter) convert dietary carnitine and dietary lecithin to TMAO

### 3.3 Faecal Metabonomic Profiles

Faecal water metabonomics were explored after feeding the pre‐trial basal haylage for 10 days and then each of the trial haylages for 12 days (Figure 3).

**Figure 3:**
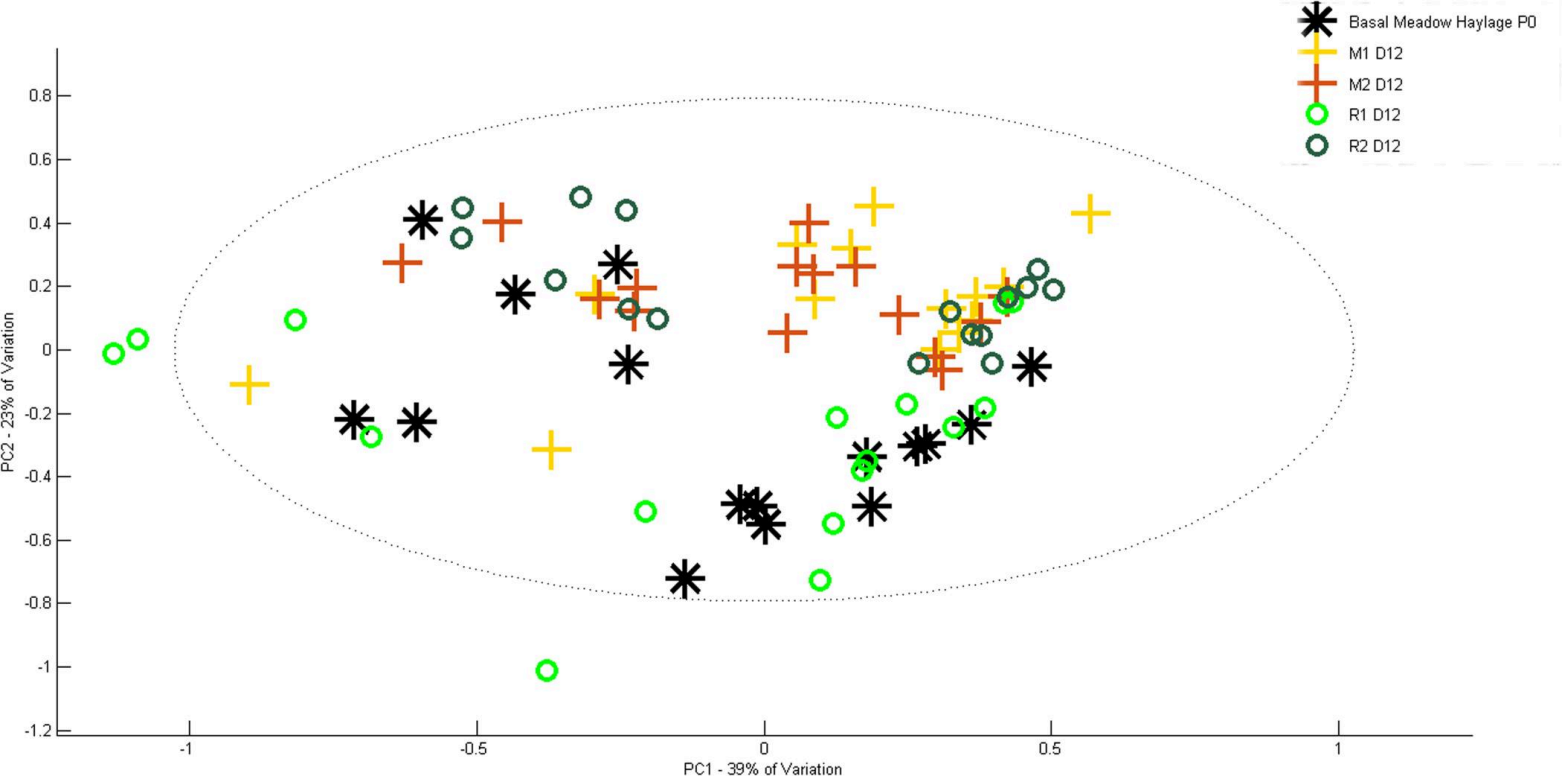
Principal component analyses (PCA) of the faecal water metabolic phenotypes of ponies fed five different haylages.[Color figure can be viewed at wileyonlinelibrary.com]

In contrast to the urinary metabonomic data, there was no clustering and no differentiation evident in the faecal metabolic phenotypes (Figure 3) showing that the faecal metabonomic output was similar when all five diets were subjected to unsupervised PCA analysed.

Metabonomic profiles of individual pony's faecal metabonomes are shown in the PCA panel plot Figure 4 and show less discriminatory patterns than the pairwise urinary profiles. When looking at the metabolites excreted on the four test haylages, some clustering was evident between R1 vs. R2 and M1 vs. R2, albeit not indicative of statistical difference. The data for R2 split into 2 distinct groups, while the two HY conserved only a week apart, i.e., R1 and M1 were very similar.

**Figure 4:**
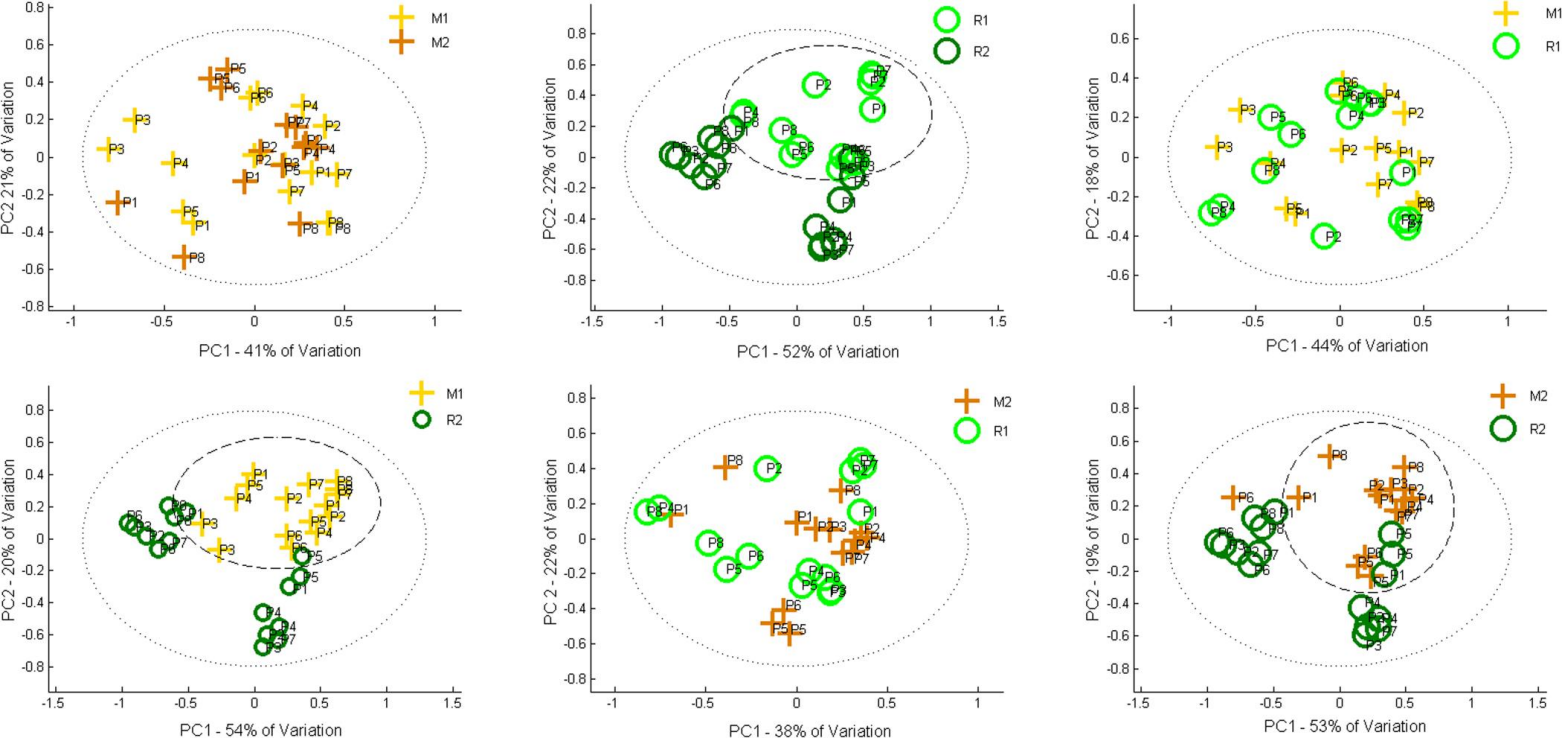
Panel plot of principal component analysis of faecal metabolic phenotypes for each pairwise comparison with each pony labeled to show individual animal effect. [Color figure can be viewed at wileyonlinelibrary.com]

The mean differences in faecal metabolites detailed in Table 4 show that all the Q^2^Y models had acceptable predictability, all being greater than 0.75. No differing individual metabolites were noted between M1 and M2 nor between M2 and R2. Pair wise comparisons between R1 and R2, M1 and R1 plus M1 and R2 as well as M2 and R1 all showed significant differences in individual metabolites, but these differences varied according to each pairwise comparison. R2 had greater amounts of acetate, malonate and methylamine compared with R1; M1 produced more p‐Hydroxyphenylpyruvate, o‐Hydroxyphenylpyruvate and 5‐Hydroxy‐l‐tryptophan than R1; M1 produced more malonate compared with R2 and M2 produced more m‐Hydroxyphenyl acetate, 5‐Hydroxytryptophan, p‐Hydroxyphenylpyruvate than R1. No common metabolic signature was present for any of the pairwise comparison, and no statistical confirmation of group separation was noted between haylages.

**Table 4:** Mean Faecal metabolites at day 12 from 4 ponies fed 4 Haylages (rye grass 1 R1, meadow 1 M1, rye grass 2 R2 and meadow 2 M2) in an in vivo total collection digestibility trial.

	Q^2^Y & Permutation *P* values	Metabolite	^1^H Resonance (δ)	Correlation coefficient (R^2^)	Predicted concentration (Mean + SD) (μM)	Concentration probability value	Function & reference to the livestock metabolome database
**M1 V M2**	0.757 *p* < 0.001	No differing metabolites					
**M2 V R2**	0.825 *p* = 0.003	No differing metabolites					
**R1 V R2**	0.792 *p* < 0.001	Acetate	1.92 (s)	0.5	R1 6.633 ± 1.555 R2 10.835 ± 1.854	0.001	LMDB00014 Short chain fatty acid
		Malonate	3.16 (s)	0.7	R1 3.196 ± 0.652 R2 5.429 ± 1.713	0.001	LMDB00217 Fatty acid biosynthesis
		Methylamine	2.61 (s)	0.6	R1 7.474 ± 1.307 R2 11.235 ± 2.190	0.001	LMDB00073 From amine catabolism
		Propionate	1.07 (t)	0.5	R1 7.503 ± 3.642 R2 6.086 ± 1.773	0.446	LMDB00110 Short chain fatty acid
**M1 V R1**	0.795 *p* < 0.001	p‐Hydroxyphenylpyruvate	6.99 (d)	0.8	M1 1.481 ± 0.645 R1 0.899 ± 0.390	0.005	LMDB00904 Phenylpyruvic acid derivatives
		o‐Hydroxyphenyl acetate	7.19 (dd)	0.7	M1 2.218 ± 0.242 R1 1.274 ± 0.626	0.003	LMDB00155 Phenolic acids
		5‐Hydroxy‐l‐tryptophan	7.28 (s)	0.7	M1 1.374 ± 0.619 R1 0.791 ± 0.381	0.004	LMDB00161 Aromatic amino acid naturally produced by the body from the essential amino acid l‐tryptophan. 5‐Hydroxy‐l‐tryptophan is the immediate precursor of the neurotransmitter serotonin.
**M1 V R2**	0.769 P0.001	Malonate	3.13 (s)	0.5	M1 12.71 ± 5.391 R2 12.32 ± 8.261	0.875	LMDB00217 Fatty acid biosynthesis
**M2 V R1**	0.831 *p* < 0.001	m‐Hydroxyphenyl acetate	6.79 (d)	0.7	M2 2.271 ± 1.379 R1 1.273 ± 0.682	0.009	LMDB00155 Phenolic acid
		m‐Hydroxyphenyl acetate	7.26 (t)	0.65	M2 1.125 ± 0.547 R1 0.776 ± 0.290	0.123	LMDB00155 Phenolic acid
		5‐Hydroxytryptophan	7.14 (s)	0.65	M2 1.401 ± 0.846 R1 0.786 ± 0.416	0.006	LMDB00161 Aromatic amino acid naturally produced by the body from the essential amino acid l‐tryptophan. 5‐Hydroxy‐l‐tryptophan is the immediate precursor of the neurotransmitter serotonin.
		p‐Hydroxyphenylpyruvate	6.99 (d)	0.7	M2 1.196 ± 0.931 R1 0.904 ± 0.445	0.016	LMDB00904 Phenylpyruvic acid derivatives
		Raffinose	5.43 (d)	0.55	M2 2.666 ± 1.071 R1 2.523 ± 1.252	0.745	LMDB00418 When members of the raffinose group of sugars are consumed by monogastrics, the animals do not have the necessary gut enzymes to digest these sugars in the intestinal tract. Consequently, they pass undigested to the large intestine where they are anaerobically fermented by gut bacteria.
		Dimethyl sulfone	3.16 (s)	0.7	M2 7.058 ± 3.928 R1 4.764 ± 2.323	0.064	LMDB00459 Derives from dietary sources, from intestinal bacterial metabolism and from animal endogenous methanethiol metabolism.

### 3.4 Apparent Digestibility

The DMI across all four haylages did not differ as it was determined by the study design; however, the Digestible energy (DE) intakes (DEI) were different, reflecting the potential degradability of each forage, with the least mature R1 haylage giving the highest daily total DE intake of 68.5 MJ/day while the more mature M2 gave the lowest at 51.5 MJ/day.

The dry matter digestibility (DMD) of R1 was also greater than for the other three haylages, despite the high degree of variability between ponies, with M1 > R2 = M2. Crude protein apparent digestibility (CPD) showed a high degree of variation between individual ponies, so analyses were done (data shown in S2) by removing a couple of the more extreme outliers and then repeated with all data included. As the results were not significantly different the decision was made to keep all the values in the analyses. As a result, no pattern according to haylage type or physiological age was seen in CPD. Neutral detergent fiber apparent digestibility (NDFD) was similar across all four feeds although the acid detergent fiber apparent digestibility (ADFD) showed that the less fibrous R1 haylage was the most digestible compared with R2 and M2, although not different from M1. No relationships were observed between pony BW and apparent digestibility, or faecal pH and feed pH, nor faecal pH and nutrient digestibility of any parameter (see Supplementary data S4).

## 4 Discussion

The hypothesis of this study that the more vegetative forage (R1) would be more digestible and generate distinct urinary and faecal metabonomic profiles compared with less digestible forages is only partially accepted. While R1 demonstrated higher DMD, this difference was not mirrored by any discernible clustering or separation in urinary or faecal profiles within the all‐feeds PCA analyses. Some discriminatory patterns did emerge in the urinary pairwise comparisons between R1 and R2 and between R1 and M1, while the faecal pairwise comparisons appeared to reflect both haylage physiological age (R1 vs. R2) and grass species (M1 vs. R1; R2 vs. M2). However, these separations were subtle and may reflect the sensitivity of multivariate methods rather than strong, biologically stimulated differences between diets.

The supplementary hypothesis that feeding a high‐energy forage would not elicit metabolites associated with compromised gut health is supported. Faecal pH was consistent across all diets, and neither a fall in pH nor elevations in metabolites linked to dysbiosis (e.g., lactate) were observed, indicating that hindgut stability was maintained throughout.

Previously published data (Weinert‐Nelsson et al. 2023; Garber et al. 2020; Mach et al. 2020) have shown that equids under a similar management and feeding regimen can have similar microbiome profiles, and the similarity of urinary metabonomic output as detailed in Figure 1 for the basal haylage at the beginning of this trial suggested this was also the case in this group of ponies. For the past 5 years the ponies were managed together as a group. Ten days prior to the trial, they were transitioned from a permanent meadow pasture to individual boxes. During this acclimatization period each pony was fed a mixed species meadow haylage, designed to prepare them for the management regimen in the study. The metabonomic profiles for the pre‐trial diet confirmed that all ponies started the trial with similar urinary metabonomic profiles with low variability between ponies, so any differences found could be attributed to the diets fed.

Three out of the four haylages fed in this study i.e., M1, M2 and R2 had fibre contents typical of mature grasses conserved for winter animal feed in UK (Sousa et al. 2021). The ADF contents of >/=306 g/kg DM and NDF contents of >/=562 g/kg DM suggest that these feeds would primarily be degraded by microbial fermentation in the large intestine of the ponies. The R1 haylage on the other hand had double the quantity of potentially digestible WSC and suggests that this proportion of the haylage would be enzymatically digested in the small intestine. The production of individual urinary and faecal metabolites detailed in Tables 3 and 4 confirms this assumption. The metabolites associated with soluble carbohydrate digestion i.e., propionyl carnitine (fatty acid transporters) and dihydroxyacetone (associated with glycolysis and a product of fructose metabolism) and of protein digestion e.g., dihydroxyacetone, dimethylamine and isoleucine, were greater for R1 compared with the other three HY. The urinary metabonome is reflective of what is digested and fermented and as the digestibility data shows the R1 haylage was more digestible than the other three haylages.

Investigating the urinary and faecal metabonomic variation via PCA models (Figures 1 and 3) from the individual pony metabolite data for each diet revealed tighter clustering in the urinary metabonome for the R1 haylage, but no such clustering was seen for any haylage in the faecal data. The tighter clustering in the urinary data may, in part, be due to a more consistent ability of all the ponies to enzymatically digest the high WSC content in the R1 haylage, supported by the higher DMD across all ponies for this diet. However, despite this tighter clustering, no differentiation was seen between any of the haylages in either the urinary or faecal metabonomic profiles. The patterns displayed for the M1, M2 and R2 diets all showed more dispersal overall compared to the R1, indicating variability within the metabonomic profiles. The fact that the more mature and mixed species M2 haylage produced more variability for the ADFD than the younger nutrient‐dense R1 haylage agrees with the findings of Gomez et al. (2021) who also reported high inter‐animal variation when high lignin diets were fed. This was not the case for the other nutrients. For DMD, OMD, CPD and NDFD the monoculture rye grass haylages (R1, R2) produced greater variability than the meadow haylages (M1, M2). This maybe a function of long‐term gut adaptation to mixed species fodder which has been the normal forage type for this group of ponies throughout their lives. However, such a high degree of individual variation across all diets might explain why the metabonomic profiles were so dispersed. Some of the variation may in part be a function of the Latin square study design which was set up to minimise any period effects. The departure from the rest of the group noted in three ponies in period one and one pony in period two could be to do with diet order, whereas two ponies moved in period one from the basal diet to R1, two from basal to R2, two from basal to M1 and two from basal to M2. Although all ponies followed the same order of diets i.e., M2 to R1 to R2 to M1 to M2, they started the sequence on different haylages, which may have influenced initial carry‐over and early microbiome profiles in those individuals.

The order of diets was used to measure the biggest difference between the haylages that owners frequently encounter e.g., the change from a more ‘hay‐like’ (M2) mature mixed‐species forage to a more vegetative monoculture nutrient‐dense R1 haylage and the differences in nutrient intake that inevitably accompanies such a change. The change from R1 to R2 and M1 to M2 were less extreme and used to measure the effect of physiological age on metabonomic production. Additionally, the study purposely did not have any ‘wash‐out’ period between diets, as one of the key factors of this study (reported in another paper from this group) was the effect on metabonomic profiles of rapid changes between diets.

As used in previous studies Nilsson et al. (2025) and Leng et al. (2021), the supervised pairwise metabonomic profiles were analysed using OPLS‐DA modelling for both urinary and faecal data. Several criteria were set to improve accuracy; the first was that goodness of fit was deemed acceptable, the second that overfitting of latent variables was not present (Gowan et al. 2010) and finally, that moderate to strong correlations were present. Differentiating urinary metabonomic cluster patterns were seen between R1‐R2 and R1‐M1 showing that herbage physiological age and species can affect the pattern of metabolic output. The profiles comparing R1‐M2 were differentiating for five ponies, but ponies 5,6 and 7 differed from the rest of the group showing profiles closer to the M2 haylage, although this particular pattern of variability was not reflected in the apparent digestibility data, for those ponies. No differentiating clustering was seen between M1‐M2, M1‐R2 or M2‐R2, suggesting that the metabonomic outputs reflected group apparent digestibility and comparable metabolic responses for these three haylages. This suggests that the younger more digestible R1 haylage, although producing variable digestibilities between ponies, supported a more stable or uniform metabolic response, likely linked to digestibility and energy metabolism in the ponies, which was also noted by Blackmore et al. (2013) when a more digestible forage was fed. Despite the high degree of individual variability, all ponies digested the R1 haylage more than the other three forages, which likely drives the more consistent urinary metabolic responses by increasing the proportion of soluble substrates available for foregut digestion.

Overall, the faecal metabonomic data (Figure 3) showed an absence of strong differentiation between diets. This is likely a reflection of the similarity of the remaining substrate entering the hindgut post foregut digestion. The larger soluble proportion (WSC) of the R1 haylage would have been digested enzymatically in the foregut, leaving the more fibrous portion of the forage to be fermented in the hindgut. This is partially supported by the lack of difference in propionate production in the pairwise analyses (Table 4) between R1 and the other 3 haylages.

Some tighter clustering was evident for the younger R1 and M1 haylages while the R2 profiles separated into 2 distinct groups with ponies, 1, 4, 5 and 7 in one cluster and 2, 3, 6 and 8 in another distinct cluster, this suggests the R2 diet caused greater metabolic variability or instability, also seen in the digestibility data.Although no common metabolic signature was present for any of the faecal pairwise comparison, and no statistical confirmation of group separation was noted, the pairwise comparison differences may be the result of the degree of difference between the nutrient content of each pair of haylages, with the maturity of the R2 driving metabolite differences when compared with the R1 while botanical composition may have driven the differences between M1 and R1. The modest degree of differentiation between the clusters in the R1‐R2 and the M1‐R2 suggests fibre maturity may have been an influencing factor in metabolite production with the more fibrous haylages producing more metabolites associated with fibre fermentation pathways i.e., acetate, malonate, and methylamine. Thus, the pairwise comparisons suggest different pathways associated with substrate type that resulted from comparing individual haylages with their own particular nutrient profile. This may have arisen from up or down regulation of different microbes in the gut (Garber et al. 2020) in response to the different nutrient proportions in each haylage.

The equid is a browsing species, and their gut microbiome is highly equipped to deal with subtle changes in dietary nutrient content due to seasonal variations in grass supply (Salem et al. 2018). The fact that no metabonomic differences were seen in the urinary data between M1, M2 and R2 shows that subtle changes in physiological age or grass species, which can slightly vary the nutrient intake, may be readily dealt with by the gut microbiome. This is largely supported by the digestibility data which showed less differences between the M1, M2 and R2 forages.

Garber et al. (2020) and Sorensen et al. (2021) have reported changes in caecal and faecal microbial communities when equids were subjected to notable differences in forage supply i.e., changes between alfalfa and grass hay, while Leng et al. (2021) have shown differences in urinary metabolites when ponies were fed hay versus haylage. Although the forage changes in this study were less extreme, as they were all haylages, the results showed that haylages with differing nutrient profiles can impact hindgut metabolic output. This reinforces the value of obtaining nutrient analyses when changing between haylages so gut microbial stability can be maintained, thus reducing the likelihood of dysbiosis caused by divergent nutrient profiles. Importantly, despite shifts in metabonomic profiles noted in this study, no metabolites indicative of dysbiosis or compromised hindgut health were detected. This could be attributed to the 3‐day changeover or diverse hindgut microbiomes that allowed the ponies to maintain gut stability despite the differing nutrient profiles in the haylages. This may have been enhanced by the fact that the four trial haylages were conserved on the same farm using the same machinery for cutting and baling, giving a degree of similarity that might not be present when haylage is conserved using different conservation techniques and equipment.

The faecal bacterial profiles were not determined in this trial, so it is not possible to determine if the different metabonomic outputs were accompanied by up or down regulation of different microbes.

Although all ponies digested the R1 > M1 > R2 = M2 the standard errors were high across all parameters, particularly for the more fibrous components of ADF in the mature R2 and M2 haylages. Such individual variation has also been noted by Antwis et al. (2018) who reported that individual variation accounted for 52.6% of the gut microbiome between individuals in a group of semi‐feral Welsh Mountain ponies. However, the high degree of individual variation did not prevent differentiation between more or less digestible diets but might explain why some animals thrive and indeed gain weight on low‐quality forages when others do not, a phenomenon noted by Morrison et al. (2020). Taken together, this study provides new insight into a previously poorly investigated forage and how haylage type and maturity shape digestive and metabolic pathways in ponies. The early cut nutrient‐dense R1 haylage did produce more distinct faecal metabolic signatures and markedly higher digestibility, while the more mature haylages had more dispersed metabolic signatures coupled with lower digestibilities, indicative of less soluble and more structural carbohydrate content. These findings highlight the need for greater recognition of haylage heterogeneity within equine nutrition and suggest that metabonomics may be a useful tool for evaluating animal responses to forage quality, informing dietary transitions and supporting individualised feeding approaches.

## 5 Conclusion

This study demonstrates that haylage botanical composition and maturity shape digestibility and influence metabolic outcomes in ponies in a meaningful and measurable way. The low‐fibre, higher‐nutritional‐value mono‐species haylage was more digestible and was associated with detectable shifts in urinary and faecal metabonomic profiles at the multivariate level. Differences in grass type or physiological age had minimal impact on overall metabonomic profiles provided nutrient content was similar. However, individual metabolite differences were detected in pairwise comparisons and reflected haylage nutrient composition, producing a unique array of differing metabolites according to each comparison.

Overall, the results support the view that horses are generally resilient to small variations in forage characteristics, with implications centred more on maintaining consistent nutrient delivery than on clinical risk. However, in high‐performance horses or those with specific clinical sensitivities, changes in nutrient availability may influence metabolic responses, underscoring the value of careful forage transitions. Haylage should not be regarded as a fully homogeneous feed category. The metabonomic distinctions observed here are indicative of underlying nutritional differences and suggest that metabolic profiling could be a useful tool for detecting how forage characteristics influence equine digestive physiology.

## Author Contributions

Study design: Meriel Moore‐Colyer, Simon Daniels, Pat Harris; Execution and data collection: Meriel Moore‐Colyer, Simon Daniels. Data interpretation and manuscript preparation: Meriel Moore‐Colyer, Simon Daniels, Pat Harris. All authors have read and agreed to the published version of the manuscript.

## Ethics Statement

The authors confirm that the ethical policies of the journal, as noted on the journal's author guidelines page, have been adhered to and the appropriate ethical review committee approval was obtained from the Royal Agricultural University Ethics Review Committee number 20204603‐Daniels. The authors confirm that they have followed EU standards for the protection of animals used for scientific purposes.

## Conflicts of Interest

The authors declare no conflicts of interest.

## Supporting information


Supporting File 1

Supporting File 2

Supporting File 3

Supporting File 4

## Data Availability

The data that support the findings of this study are available from the corresponding author upon reasonable request.
